# Transarterial chemoembolization combined with atezolizumab plus bevacizumab conversion therapy for intermediate-stage hepatocellular carcinoma: a case report and literature review

**DOI:** 10.3389/fimmu.2024.1358602

**Published:** 2024-05-28

**Authors:** Haidong Ai, Ting Gong, Yongbiao Ma, Guixu Ma, Wei Ding, Weibao Ding, Wenjuan Wang, Xuelin Zhao

**Affiliations:** ^1^ Hepatobiliary and Pancreatic Medical Center, The First Affiliated Hospital of Weifang Medical College (Weifang People’s Hospital), Weifang, China; ^2^ Department of Ophthalmology, The First Affiliated Hospital of Weifang Medical College (Weifang People’s Hospital), Weifang, China

**Keywords:** atezolizumab plus bevacizumab, transarterial chemoembolization, intermediate-stage unresectable hepatocellular carcinoma, conversion therapy, immunotherapy

## Abstract

Hepatocellular carcinoma (HCC) ranks as the sixth most common malignancy globally, with the majority of patients presenting at the initial diagnosis with locally advanced or metastatic disease, precluding the opportunity for curative surgical intervention. With the exploration and advancement of locoregional treatments, novel molecular-targeted therapies, anti-angiogenic agents, and immunomodulatory drugs, the management of HCC has seen an increase in objective response rates and prolonged duration of response significantly enhancing the potential for conversion to resectable disease in intermediate and advanced-stage unresectable HCC. Herein, we present a case of Barcelona Clinic Liver Cancer stage B unresectable HCC, where after two courses of treatment with transarterial chemoembolization combined with atezolizumab plus bevacizumab significant tumor reduction was achieved. Per Response Evaluation Criteria in Solid Tumors 1.1, partial response culminated in successful curative surgical resection. No drug-related adverse reactions occurred during hospitalization, and there has been no recurrence during the 11-month postoperative follow-up. For patients with Barcelona Clinic Liver Cancer stage B (intermediate-stage) unresectable HCC, the transarterial chemoembolization combined with atezolizumab plus bevacizumab regimen may offer improved therapeutic outcomes leading to a higher success rate of conversion therapy and, thus, improved survival.

## Introduction

HCC remains one of the most prevalent malignancies globally ranking sixth in incidence and third in cancer-related mortality (1). Of patients, 60%–70% are diagnosed with unresectable HCC (2, 3). For such patients, administering systemic and/or locoregional treatment to create an opportunity for radical resection, reduce postoperative recurrence, and improve prognosis is referred to as conversion therapy for liver cancer (4). Tumor reduction or downstaging following conversion therapy is a crucial means for unresectable HCC patients to achieve curative resection and long-term survival. Historically, conversion therapy has primarily involved local treatments, with a low conversion resection rate and overall poor efficacy. However, with the emergence and advancement of various molecularly targeted drugs and immune checkpoint inhibitors (ICIs), systemic therapy has rapidly progressed, leading to a gradual increase in conversion resection rates, significantly improving the prognosis of intermediate and advanced-stage HCC patients (2, 5, 6).

The clinical guidelines from the European Association for the Study of the Liver (EASL) and the American Association for the Study of Liver Diseases (AASLD) recommend transarterial chemoembolization (TACE) and atezolizumab plus bevacizumab (Atezo/Bev) as the first-line treatment choice for Barcelona Clinic Liver Cancer (BCLC) stage B (intermediate-stage) HCC patients (7, 8). However, to date, there have been no studies reporting the survival outcomes of intermediate-stage HCC patients undergoing curative surgery following TACE combined with Atezo/Bev conversion therapy. This article reports on the detailed treatment process of a case of large unresectable HCC classified as BCLC stage B, which underwent only two sessions of TACE combined with the Atezo/Bev regimen, resulting in significant tumor volume reduction and subsequent successful radical surgical resection. By presenting this case and combining it with relevant literature, we aim to explore new treatment approaches for similar patients and provide a reference for future clinical endeavors.

## Case presentation

**Figure 1:**
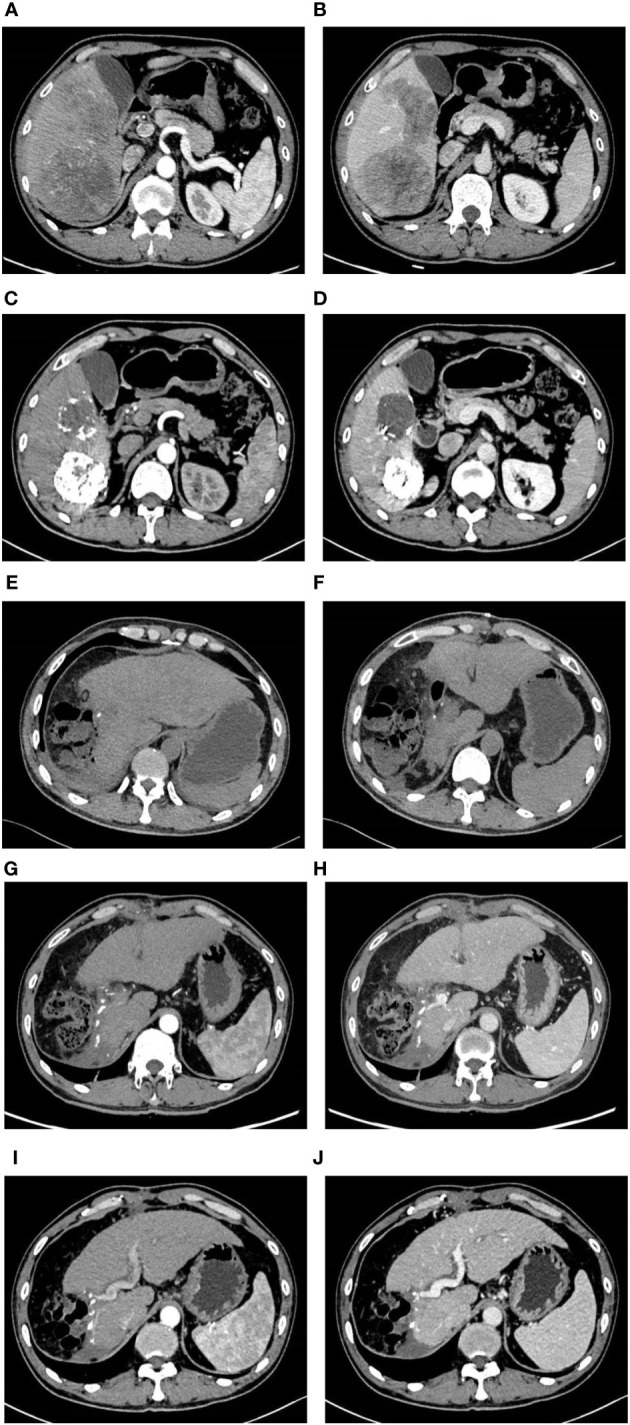
Imaging pictures. Before TACE combined with Atezo/Bev treatment, the abdomen enhanced CT revealed multiple tumors in the right liver, with the largest measuring approximately 10 cm × 7.50 cm. During the arterial phase, the tumors exhibited uneven enhancement, while during the venous phase, the degree of enhancement was relatively reduced **(A, B)**. Following two courses of TACE combined with Atezo/Bev treatment, the abdomen enhanced CT indicated a significant reduction in the size of the right liver tumors compared to before, with the largest measuring approximately 6.9 cm × 6.2 cm. Patchy high-density shadows were observed internally, considered to be iodine oil deposition after TACE treatment, with no obvious enhancement on the enhanced scan **(C, D)**. Three days after liver resection, the abdomen plain CT indicated postoperative changes in the right liver lobe, with a small amount of free gas around the liver and no significant fluid accumulation **(E, F)**. One month after liver resection, the abdomen enhanced CT revealed postoperative changes in the right liver lobe, with no abnormal enhanced lesions in the liver parenchyma and a small amount of abdominal fluid **(G, H)**. Six months after liver resection, the abdomen enhanced CT indicated the absence of the right liver lobe, with no abnormal enhanced lesions in the liver parenchyma and a small amount of abdominal fluid around the liver **(I, J)**.

**Figure 2:**
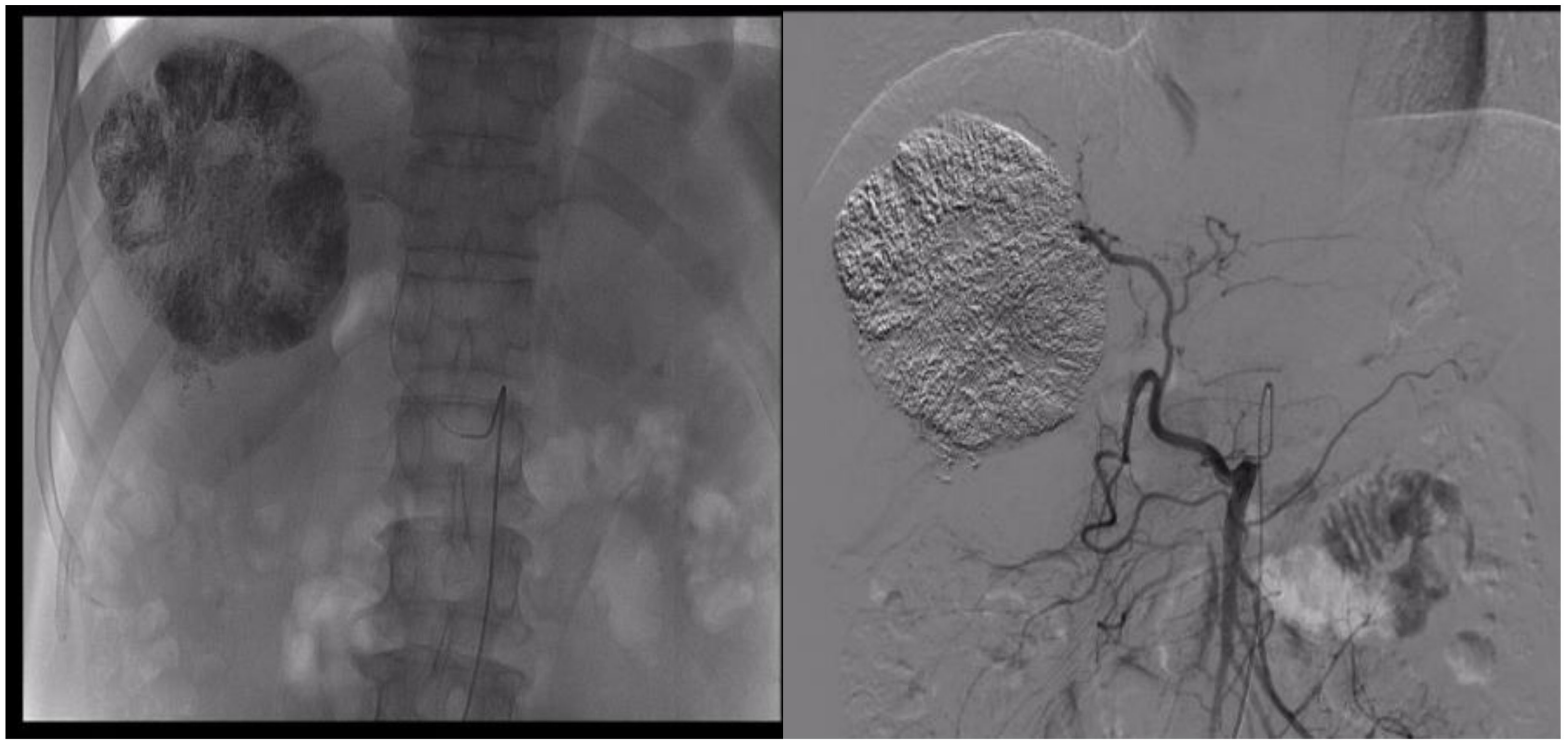
During the TACE procedure, the imaging showed staining of the right liver tumors.

The patient, a 53-year-old male, was admitted to the hospital on 13 March 2023 due to persistent nausea for 2 months and the discovery of a liver mass 10 days prior. He has experienced fatigue and weight loss over the past 2 months. His medical history includes a 2-year duration of hepatitis B without regular antiviral treatment. There was no history of hypertension, diabetes, or coronary artery disease, and no family history of psychological disorders or genetic conditions. Before the current visit, he had not received any treatment. Physical examination revealed tenderness in the right upper abdomen, with no other abnormalities noted. Laboratory tests revealed elevated levels of AFP (45,316.0 ng/ml, normal range 0–10 ng/ml), AFP-L3% (24.1%, normal range 0%–10%), PIVKA-II (11,278.0 ng/ml, normal range 0–40 ng/ml), HBV-DNA (2.386 × 103 IU/ml, normal range <500 IU/ml), ALT (69 U/L, normal range 0–50 U/L), AST (65 U/L, normal range 0–40 U/L), GGT (135 U/L, normal range 4–60 U/L), T-BIL (45.8 µmol/L, normal range 0–23 µmol/L). The preoperative indocyanine green (ICG) retention rate at 15 min was 12%. Routine blood examination and coagulation function showed no significant abnormalities. Enhanced CT of the upper abdomen revealed multiple liver lesions, with the largest measuring 10 cm in diameter consistent with HCC and multiple intrahepatic metastases (**Figures 1A, B**). Considering the medical history, serological markers, and imaging examinations, intrahepatic cholangiocarcinoma and benign liver tumors were excluded. The preoperative diagnosis was HCC, BCLC stage B, Child–Pugh class A, and Eastern Cooperative Oncology Group (ECOG) Performance Status 0. Following a multi-disciplinary discussion, we identified three tumors in the patient, with the largest lesion measuring approximately 10 cm in diameter and the combined diameter of the two largest lesions totaling approximately 18.2 cm. The lesions predominantly affected the liver S5, 6, and 7, with suspected tumor invasion into S8. If a right hepatectomy was performed, the estimated residual liver area would be 38%. Considering the patient’s concurrent liver cirrhosis, which complicates extensive liver resection, we deemed the tumor non-resectable, necessitating conversion therapy to create a surgical opportunity. Hence, we devised a treatment regimen consisting of the TACE combination with Atezo/Bev for the patient. The TACE treatment was to be administered every 4 weeks, while the Atezo/Bev treatment was to be administered every 3 weeks. Concurrently, oral entecavir antiviral therapy was initiated, and the patient and their family members were thoroughly informed about the potential complications and adverse reactions of TACE combination with Atezo/Bev therapy, as well as the possibility of tumor progression despite treatment. Additionally, the necessity of surgical resection following successful conversion was conveyed. The patient’s family members expressed consent and agreed to the treatment plan. The pharmaceuticals administered for the TACE procedure comprised 4 mg of raltitrexed and 20 mg of pirarubicin. The treatment process was as follows: Hepatic arterial angiography revealed tumor staining, with the catheter inserted into the hepatic artery. Initially, 4 mg of raltitrexed was administered, followed by selective insertion of a microcatheter into the tumor-feeding arteries, delivering 10 ml of iodized oil plus 20 mg of pirarubicin, effectively disrupting the tumor’s blood supply. Subsequent imaging revealed satisfactory deposition of the iodized oil (**Figure 2**). The patient experienced mild fever on the first day after the procedure, without any other adverse reactions. Two days after receiving TACE treatment, the Atezo/Bev therapy was initiated, involving an intravenous infusion of atezolizumab (1,200 mg) and bevacizumab (15 mg/kg), with no drug-related adverse reactions observed during the treatment process.

**Figure 3:**
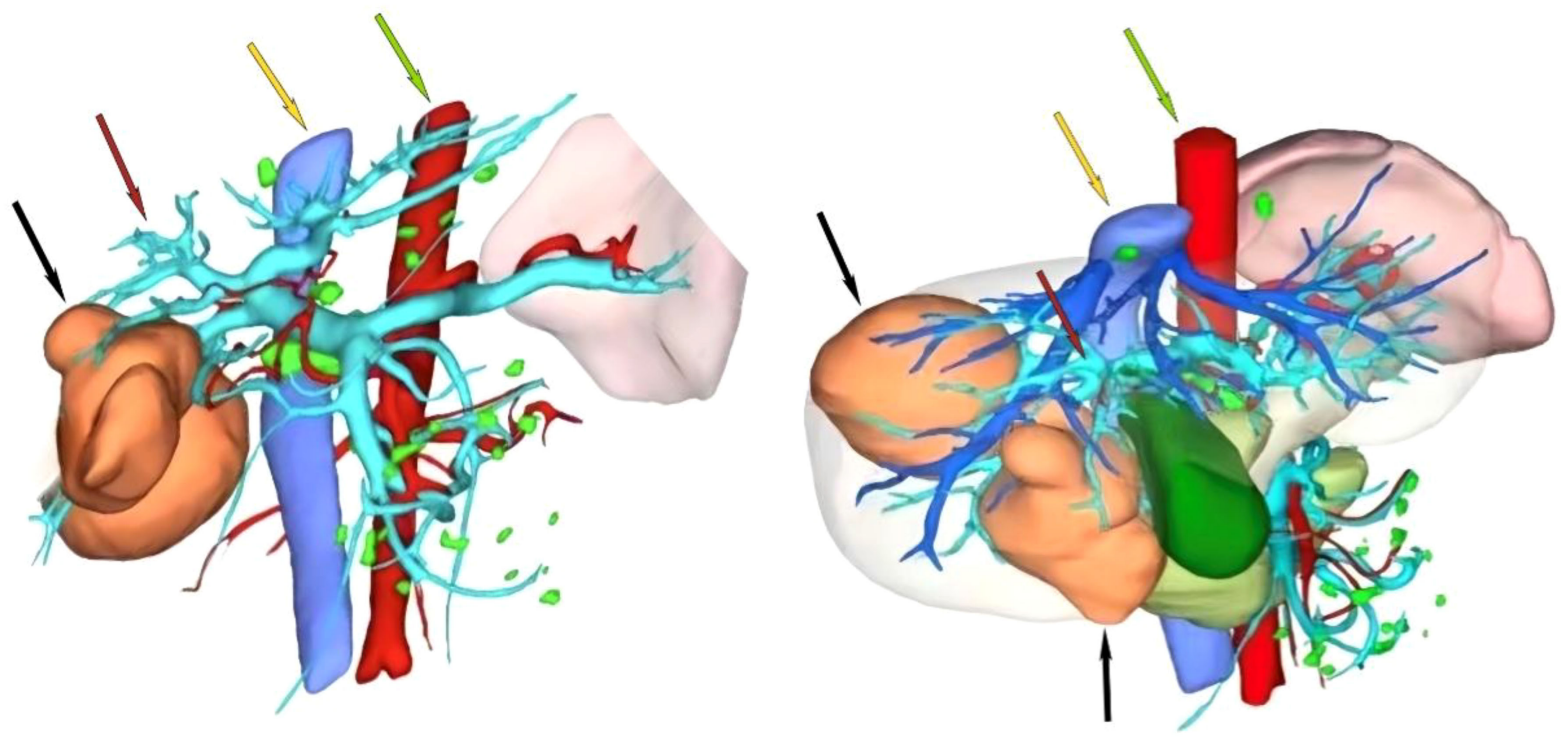
Preoperative CT three-dimensional reconstruction model. The black arrow indicates the tumor, the red arrow indicates the portal vein, the yellow arrow indicates the inferior vena cava, and the green arrow indicates the abdominal aorta.

Following two cycles of TACE combined with Atezo/Bev therapy, a subsequent examination revealed significant reductions in AFP (1,710.0 ng/ml, normal range 0–10 ng/ml), AFP-L3% (19.35%, normal range 0–10%), and PIVKA-II (261.3 ng/ml, normal range 0–40 ng/ml). Abdomen-enhanced CT displayed marked tumor shrinkage, with the maximum tumor diameter reducing to 6.9 cm, and the diameter of the two largest lesions decreasing to 12.3 cm, demonstrating a reduction exceeding 30% compared to the initial assessment (**Figures 1C, D**). According to the Response Evaluation Criteria in Solid Tumors 1.1 (RECIST 1.1), the treatment response was assessed as a partial response (PR). Subsequent multi-disciplinary discussion determined that post-conversion therapy, there was a notable diminution in tumor volume facilitating the preservation of S8 hepatic tissue. At this juncture, the estimated remnant liver volume reached 58.9% allowing for safe implementation of R0 resection and with more than a 2-week interval from the last Atezo/Bev treatment. Subsequently, comprehensive communication with the patient and their family was conducted providing detailed information regarding the potential, necessity, risks, and complications of the surgical resection to which the patient and family provided informed consent. Before the procedure, a comprehensive CT three-dimensional reconstruction model (**Figure 3**) was perfected, and on 4 May 2023, a laparoscopic hepatic partial resection was performed.

**Figure 4:**
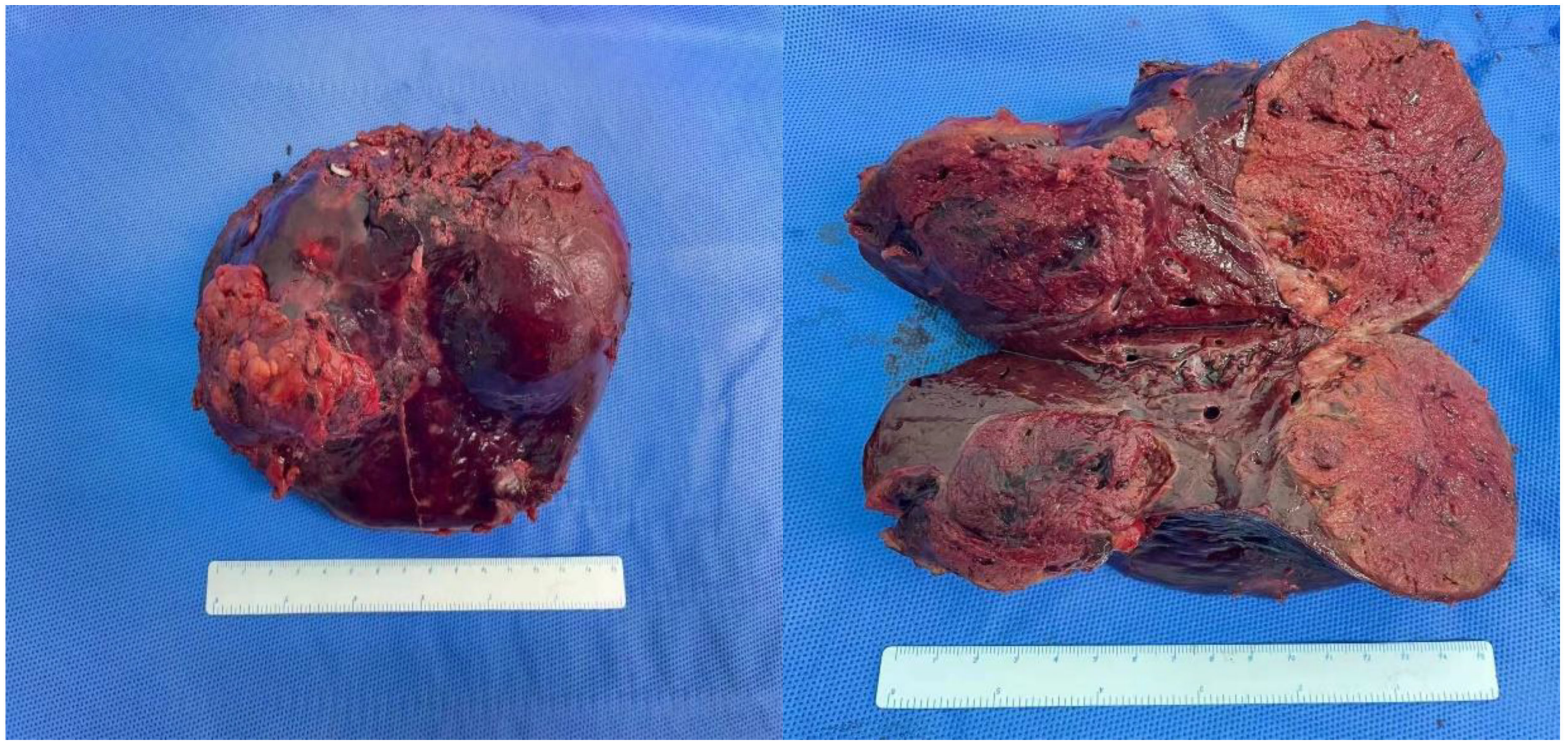
Specimen of tumor resection. Resection specimen of the tumor: there were a total of three tumors, with volumes of 6.9 cm × 6.2 cm × 6 cm, 5 cm × 4 cm × 3.8 cm, and 2.2 cm × 1.8 cm × 1.8 cm, respectively.

**Figure 5:**
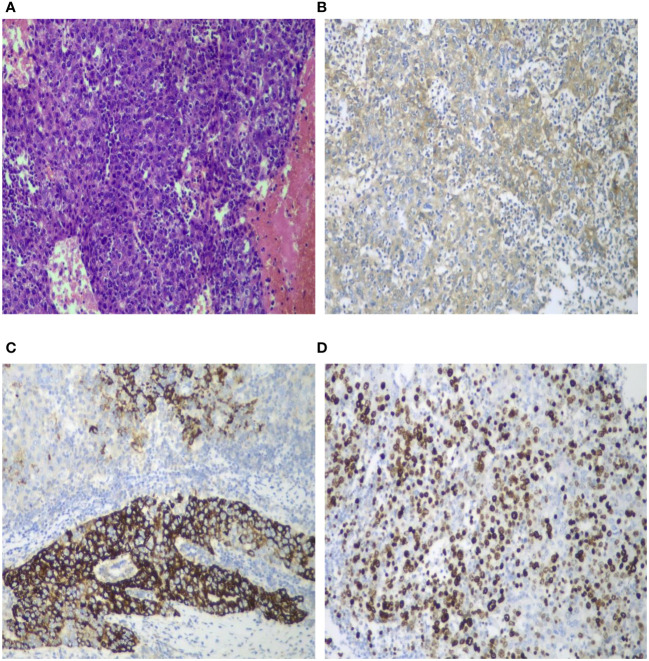
Pathological and immunohistochemical images. Histological staining of the tumor sections (HE ×100) revealed poorly differentiated hepatocellular carcinoma accompanied by extensive degeneration and necrosis. The tumor cells exhibited significant atypia, including the presence of giant cells, without satellite nodules, definite neural invasion, or intravascular tumor thrombi. Nodular cirrhosis was observed around the liver section, with partial hepatocyte cholestasis and fatty degeneration **(A)**. AFP demonstrated a positive reaction **(B** ×100**)**. HepPar-1 exhibited partial positive reaction **(C** ×100**)**. Ki-67 positivity index was 60% **(D** ×100**)**.

**Figure 6:**
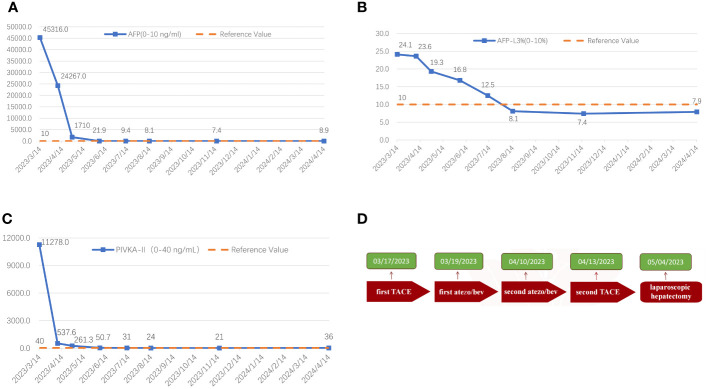
The graphical depiction of the dynamic shifts in AFP **(A)**, AFP-L3% **(B)**, and PIVKA-II **(C)** throughout the therapeutic course, along with the treatment timeline **(D)**.

The surgical procedure proceeded as follows: meticulous exploration of the abdominal cavity under laparoscopy revealed no evidence of tumor metastasis. Intraoperatively, ultrasonography confirmed the presence of the tumor in liver segments S5, 6, and 7. Considering the patient’s hepatitis B-related cirrhosis, a decision was made to preserve liver segment S8 to protect hepatic function. The tumor and liver S5, 6, and 7 were completely excised. The duration of the surgery was 3.5 h with an estimated intraoperative blood loss of approximately 150 ml. Postoperative pathological examination revealed poorly differentiated HCC, and extensive necrosis (approximately 70%) was observed within the tumors, with three nodular tumors measuring 6.8 cm × 6.2 cm × 6 cm, 5 cm × 4 cm × 3.8 cm, and 2.2 cm × 1.8 cm × 1.8 cm (**Figure 4**). The tumors did not invade the capsule, and no satellite nodules, definite neural invasion, or intravascular cancer thrombi were observed. No tumor tissue was found at the resection margins; nodular cirrhosis was observed surrounding the margins (**Figure 5A**). Immunohistochemical staining revealed AFP (+), HepPar-1 partial (+), Ki-67 index 60% (**Figures 5B–D**). An upper abdomen CT conducted 3 days postoperatively revealed the postoperative appearance of the right lobe of the liver, with no significant fluid accumulation in the abdominal cavity or around the liver (**Figures 1E, F**). The abdominal drainage tube was removed 1 week after the surgery, and the patient was discharged uneventfully after 10 days. One month postoperatively, tumor-related markers indicated AFP (21.9 ng/ml, normal range 0–10 ng/ml), AFP-L3% (16.84%, normal range 0%–10%), and PIVKA-II (50.7 ng/ml, normal range 0–40 ng/ml), with no apparent abnormalities on upper abdomen enhanced CT (**Figures 1G, H**). Over the subsequent 11-month follow-up period, tumor markers remained within normal limits (**Figures 6A–C**), and no tumor recurrence was observed in the imaging examination after the surgery (**Figures 1G–J**). Relevant data and the treatment timeline are depicted in **Figure 6**.

## Discussion

HCC is the most prevalent malignant tumor of the liver accounting for 75% to 85% of primary liver cancer cases. With over 500,000 new cases annually, approximately 80% of HCC patients are unable to undergo curative surgical treatment due to reasons such as local tumor invasion, distant metastasis, or inadequate residual liver volume (1, 3, 9, 10). In such instances, local and systemic treatments have emerged as the standard therapeutic approaches for intermediate and advanced-stage HCC. The purpose of conversion therapy is to convert unresectable HCC into resectable HCC playing a crucial role in improving the prognosis of intermediate and advanced-stage HCC (11–14).

In recent years, groundbreaking progress has been made in researching novel anti-angiogenic agents and ICIs. These two combined applications can enhance host immune activity and tumor immunogenicity synergistically exerting antitumor effects and achieving favorable therapeutic efficacy in patients with intermediate and advanced-stage HCC. Bevacizumab is an anti-angiogenic agent that selectively binds to vascular endothelial growth factor (VEGF) blocking its bioactivity and inhibiting the binding of VEGF to the VEGFR-1 and VEGFR-2 on endothelial cells, thereby reducing tumor angiogenesis and inhibiting tumor growth (15). Atezolizumab is a programmed death-ligand 1 (PD-L1) inhibitor that reverses T-cell-mediated tumor immune evasion by binding to PD-L1 on tumor cells and blocking its interaction with T cells and PD-1 promoting T-cell attack tumor cells and exerting antitumor effects (16). The global, multicenter Phase III clinical trial results of IMbrave150 (17), published in 2020, demonstrated that the Atezo/Bev significantly prolongs the overall survival of patients with intermediate and advanced-stage HCC, with a 12-month survival rate of up to 67.2% and a 42% reduction in the risk of death significantly higher than the clinical efficacy of sorafenib. Thus, the Atezo/Bev regimen has become the first-line systemic treatment for unresectable HCC. However, the sole use of Atezo/Bev typically fails to achieve sustained clinical complete response (CR) or drug-free status, with most cases experiencing progression within 1–1.5 years even with continuous treatment (2). In 2023, Cao et al. (18) reported a clinical study of TACE combined with Atez/Bev versus sole Atez/Bev treatment for advanced HCC patients. Compared to sole Atez/Bev treatment, the combination therapy of TACE with Atez/Bev demonstrated superior overall survival (OS), progression-free survival (PFS), and objective response rate (ORR) for advanced HCC patients, with reliable safety. TACE, as the first-line locoregional treatment for intermediate-stage HCC patients, although demonstrated good short-term efficacy did not lead to a satisfactory long-term prognosis, with a 3-year survival rate of only 26% (19–23). In recent years, the application of novel anti-angiogenic drugs and ICIs in the treatment of HCC has led to higher ORR and longer duration of response (DOR). Combination systemic therapies based on TACE are increasingly being utilized for patients with unresectable HCC who have the potential for conversion. Therefore, combining TACE with the Atezo/Bev regimen has become a new research direction for patients with intermediate HCC in current research.

For patients with intermediate-stage and advanced HCC who have failed first-line treatment, the second-line therapeutic approach becomes particularly crucial. In recent years, numerous second-line therapies have been introduced to treat HCC, among which several combination therapy regimens have yielded promising outcomes. A meta-analysis (24) focusing on second-line treatment for advanced HCC involved 5,488 patients and evaluated 12 treatment regimens. The findings indicated that regorafenib and cabozantinib demonstrated favorable safety and efficacy in individuals resistant to sorafenib, thus serving as viable options for second-line therapy in advanced HCC. Additionally, ICIs have also been examined as novel second-line agents in the treatment of HCC. They included nivolumab (alone or in combination with ipilimumab) and pembrolizumab, two monoclonal antibodies that block the PD-1 pathway and have been approved by the FDA for already treated HCC, following promising results in the CheckMate 040 and KEYNOTE-224 clinical trials, further broadening the treatment landscape (25–27).

Immune-combined TACE therapy remains in the exploratory phase. The IMMUTACE study (28) from Germany in 2020 is one of the earliest research studies to combine TACE with an ICI (nivolumab), involving 49 patients with intermediate-stage HCC, achieving an ORR of 71%. With a median follow-up of 14.6 months, the median PFS reached 6.14 months, and the median overall survival (OS) was 28.32 months meeting its primary endpoints and confirming the safety and efficacy of TACE in combination with ICIs. Various combination therapy regimens primarily involving TACE are continuously being explored, among which the triplet therapy combining TACE with anti-angiogenic drugs and ICIs is considered one of the most promising treatment options. The EMERALD-1 study (29) is the first global multicenter phase III clinical trial to demonstrate positive results of TACE combined with ICIs and anti-angiogenic drugs for locally advanced HCC, and it was reported at the ASCO GI 2024 congress. This study aimed to explore the effectiveness and safety of TACE combined with durvalumab with or without bevacizumab for locally advanced HCC. Analysis of the 616 enrolled patients showed that the median PFS for the TACE combined with durvalumab and bevacizumab group was 15 months, significantly improving PFS compared to the TACE-alone group (8.2 months), reducing the risk of death by 23% compared to sole TACE treatment. Several phase III clinical trials are currently underway. The LEAP-012 study (30) will assess the clinical efficacy of TACE combined with lenvatinib and pembrolizumab for intermediate-stage unresectable HCC patients (NCT04246177). The CheckMate74W trial aims to explore the efficacy and safety of TACE combined with nivolumab with or without ipilimumab for intermediate-stage unresectable HCC (NCT04340193). Both studies are ongoing, and we anticipate achieving encouraging positive results compared to sole TACE treatment.

Relying solely on TACE treatment make it challenging to achieve favorable therapeutic effects. Previous studies have indicated that TACE can lead to increased expression of HIF-1, inducing upregulation of VEGF and platelet-derived growth factor, further promoting angiogenesis and ultimately resulting in tumor recurrence (31, 32). Bevacizumab can enhance the efficacy of TACE by normalizing tumor vessels through the regulation of VEGF, thus improving drug delivery concentration (15). Therefore, combining TACE and bevacizumab can reduce post-TACE angiogenesis in HCC patients, thereby delaying tumor progression and enhancing treatment efficacy. On the other hand, TACE can induce tumor cell necrosis and release tumor-specific antigens, while promoting the recruitment and activation of dendritic cells in the microenvironment, transforming the immunosuppressive microenvironment into an immune-supportive one. Once combined with immunotherapy, TACE can enable immune checkpoint inhibitors to function better in the activated microenvironment achieving favorable therapeutic effects (33, 34). Therefore, the theoretical synergistic effects of TACE combined with Atezo/Bev may maximize the anticancer effects and effectively enhance the success rate of conversion therapy.

In the IMbrave150 study, the Atezo/Bev regimen appeared to demonstrate more precise therapeutic effects in patients with intermediate-stage (BCLC-B) HCC exhibiting a significantly longer overall survival and progression-free survival compared to advanced-stage HCC (25.8 vs. 17.5 months and 12.6 vs. 6.5 months, respectively). As per RECIST 1.1, the objective response rate for intermediate-stage HCC patients receiving Atezo/Bev systemic treatment is 44%, while it is 27% for advanced-stage HCC patients. This suggests that the Atezo/Bev regimen has a considerable tumor shrinkage effect for intermediate-stage HCC patients potentially offering a more advantageous curative conversion for these patients (35). Kudo et al. (36) conducted a multicenter study of Atezo/Bev regimen treatment for intermediate-stage HCC patients unsuitable for TACE and unresectable, enrolling a total of 110 BCLC-B stage HCC patients. Following systemic treatment with the Atezo/Bev regimen, 38 patients (35%) achieved clinical CR (comprising 7 surgical cases, 13 ablation cases, 15 TACE cases, and 3 cases using Atezo/Bev alone). Among these 38 patients, 25 reached drug-free status (7 surgical cases, 8 ablation cases, and 10 TACE cases), while the remaining 13 patients did not meet the drug-free criteria. During a median observation period of 21.2 months, the 25 patients in the drug-free status did not experience recurrence, whereas among the 13 patients who continued medication, 3 experienced a recurrence. Therefore, achieving a drug-free status can significantly delay tumor progression and prevent tumor recurrence. In this study, it was observed that all seven patients who underwent sequential surgical resection after Atezo/Bev treatment achieved a drug-free status and did not experience tumor recurrence during a median follow-up period of 21.2 months. This, to a certain extent, suggest that curative surgical resection following successful conversion is an extremely effective treatment strategy for preventing tumor recurrence, and achieving a pathological CR could provide these patients with longer disease-free survival and overall survival. However, Kudo’s study (36) only evaluated the efficacy of Atezo/Bev therapy in intermediate-stage unresectable HCC patients, and there is currently no internationally reported survival data for curative conversion outcomes of TACE combined with Atezo/Bev in unresectable HCC patients. Theoretically, the amalgamation of both treatments demonstrate a synergistic antineoplastic impact potentially maximizing efficacy and enhancing conversion success rates. In China, Wang et al. (37) conducted a multicenter retrospective study that included 21 patients with intermediate-stage HCC beyond Up-to-Seven Criteria who received TACE combined with Atezo/Bev treatment from March to September 2021. The median follow-up time was 11.7 months, with a best objective response rate (ORR) of 42.9% and a disease control rate (DCR) of 100%. In this study, TACE combined with Atezo/Bev demonstrated promising efficacy and acceptable safety potentially becoming a novel treatment option for intermediate-stage HCC patients. Another multicenter phase II clinical trial called DEMAND (38) is currently ongoing assessing the safety and effectiveness of TACE combined with Atezo/Bev treatment in intermediate-stage unresectable HCC patients. We look forward to the final experimental results of this clinical study.

We present a case of successful R0 resection following therapy involving a combination of TACE and Atezo/Bev regimen in a patient with giant BCLC-B stage (intermediate-stage) unresectable HCC. Due to the multifocality and large volume of the tumor, achieving R0 resection at stage I posed significant challenges. Even with surgical intervention, issues such as insufficient volume of the remaining liver, impaired liver function, and high risk of postoperative recurrence persisted. Remarkably, this patient exhibited significant tumor shrinkage after just two sessions of TACE combined with the Atezo/Bev regimen achieving a clinical PR and ultimately undergoing successful laparoscopic R0 resection. Eleven months postoperatively, no tumor recurrence was observed, and the patient’s quality of life remained good. This approach may offer a curative opportunity for a larger cohort of intermediate-stage unresectable HCC patients potentially leading to long-term survival. This reported sample size is limited, which poses certain constraints. Currently, there is a paucity of clinical research on the conversion therapy of unresectable intermediate-stage HCC with this regimen, and its efficacy still necessitate further confirmation through prospective multicenter large-scale randomized controlled trials.

## Conclusion

In summary, intermediate-stage HCC represents a potentially curable ailment, with the treatment objective being the attainment of clinical/pathological CR and drug-free status. The combination of TACE with Atezo/Bev regimen may potentially offer improved therapeutic efficacy and conversion resection rates for patients with intermediate-stage unresectable HCC. Upon achieving significant tumor reduction during treatment, a proactive transition to curative surgery can maximize the prognosis of such patients.

## Data availability statement

The original contributions presented in the study are included in the article/supplementary material. Further inquiries can be directed to the corresponding author.

## Ethics statement

Written informed consent was obtained from the individual(s) for the publication of any potentially identifiable images or data included in this article.

## Author contributions

HA: Writing – original draft. TG: Writing – review & editing. YM: Writing – review & editing. GM: Writing – review & editing. WD: Writing – review & editing. WBD: Writing – review & editing. WW: Writing – review & editing. XZ: Writing – review & editing.
